# The first study on urinary loss of iron and transferrin in association with proteinuria in dogs with chronic kidney disease

**DOI:** 10.14202/vetworld.2023.154-160

**Published:** 2023-01-26

**Authors:** Nawat Sannamwong, Chollada Buranakarl, Saikaew Sutayatram, Monkon Trisiriroj, Thasinas Dissayabutra

**Affiliations:** 1 Residency Program in Internal Medicine, Faculty of Veterinary Science, Chulalongkorn University, Bangkok 10330, Thailand; 2 The Small Animal Teaching Hospital, Faculty of Veterinary Science, Chulalongkorn University, Bangkok, 10330, Thailand; 3 Department of Physiology, Faculty of Veterinary Science, Chulalongkorn University, Bangkok, 10330, Thailand; 4 STAR Unit of Renal Biochemistry and Stone Disease, Department of Biochemistry, Faculty of Medicine, Chulalongkorn University, Bangkok, 10330, Thailand

**Keywords:** dog, iron, proteinuria, total iron-binding capacity, transferrin

## Abstract

**Background and Aim:**

Anemia is an important factor in surviving chronic kidney disease (CKD). Anemia in CKD is associated with various factors, such as inadequate production of erythropoietin and the availability of iron and its binding protein. Reduced total iron-binding capacity (TIBC) and iron concentrations may be related to their urinary loss along with proteinuria. This study aimed to determine the urinary loss of iron and transferrin (TF) in relation to the degree of proteinuria.

**Materials and Methods:**

The study was performed on 37 dogs with CKD. Dogs were divided according to the severity of proteinuria into two groups based on the mean of urinary protein–creatinine (UPC) ratio into UPC ratio <4 and UPC ratio >4. The hematocrit (HCT), blood chemistries, plasma iron, plasma TF, UPC ratio, urinary iron per creatinine ratio (U-Iron/CR), and urinary TF per creatinine ratio (U-TF/CR) were evaluated.

**Results:**

Anemia was associated with the severity of renal impairment as demonstrated by reduction of HCT when staging of CKD was higher. Dogs with UPC ratio >4 had higher urinary loss of both U-Iron/CR (p < 0.01) and U-TF/CR (p < 0.001) with lower plasma TIBC (p < 0.001). The UPC ratio was positively correlated with both U-Iron/CR (r = 0.710, p < 0.001) and U-TF/CR (r = 0.730, p < 0.001) but negatively with TIBC (r = –0.462, p < 0.01).

**Conclusion:**

Proteinuria was associated with urinary loss of both iron and TF which may contribute to anemia in CKD.

## Introduction

Both anemia and proteinuria were associated with decreased survival rate in dogs [1, 2] and cats [3, 4]. Non-regenerative anemia usually presents in the advanced stage of chronic kidney disease (CKD), which is associated with inadequate production of erythropoietin (EPO) [5] due to the loss of functional renal parenchyma. However, other factors may contribute to the anemia in CKD, such as uremic toxin, inflammation, nutritional imbalance, blood loss, disordered iron metabolism, or shortened erythrocyte survival [6–8]. In patients with CKD, iron deficiency could be developed from decreased consumption, reduced gastrointestinal absorption, loss through gastrointestinal bleeding, or urinary loss through glomerular filtration. Measurement of the iron panel analysis, which includes serum iron concentration, ferritin, and total iron-binding capacity (TIBC), is available in clinical practice. Normally, serum iron concentration is mostly bounded to transferrin (TF), an iron transport protein, while their interaction was reviewed earlier [9]. The TF level and saturation can be used to indicate the iron status [10, 11], while it can be estimated indirectly as TIBC using a summation of serum iron concentration and unsaturated iron-binding capacity (UIBC) [11]. Measurement of the iron panel may be essential since altered iron homeostasis could play an important role in impaired erythropoiesis in patients with CKD particularly when EPO is still sufficient.

The previous study showed that erythroid hypoplasia and normocytic normochromic anemia were common in dogs with CKD with a prevalence of >80%, and serum iron level showed significant correlations with erythrocyte precursors from bone marrow aspirates in dogs with early stages of CKD but not in the late stage [12]. However, serum EPO in those dogs showed no correlation with bone marrow or erythrogram findings. For anemia treatment response in dogs with CKD, baseline hematocrit (HCT), iron supplementation, dosage of darbepoetin, severity of azotemia, age, or comorbidities of dogs showed no correlation with darbepoetin treatment responses [13]. While in another study, low iron and TIBC were associated with the degree of anemia and the erythropoietic response to darbepoetin treatment with iron supplementation in dogs with CKD [14]. Thus, alteration in iron metabolism and bone marrow status could significantly impact erythropoiesis in dogs with CKD, even with EPO treatment and iron supplementation. Proteinuria and alteration in iron status have been demonstrated in patients with Type 2 diabetes mellitus and nephrotic syndrome, and iron deficiency was reported to be approximately 33% in children with nephrotic syndrome [15, 16]. Excessive urinary losses of iron, TF, EPO, transcobalamin, and/or copper in relation to anemia and its ineffectiveness after iron and EPO supplementation in nephrotic syndrome were extensively reviewed [17]. It was also suspected that iron and TF may be lost, especially in proteinuric dogs, which may in part, be responsible for anemia in these dogs with CKD. Increased urinary excretions of both albumin (ALB) and TF were previously demonstrated in Stage I CKD cats [18].

Therefore, this study aimed to evaluate the effect of proteinuria on urinary loss of iron and TF and plasma TIBC levels in proteinuric dogs. This is the first study regarding the proteinuria on urinary loss of iron or iron-binding protein which may affect TIBC in dogs with CKD.

## Materials and Methods

### Ethical approval and informed consent

The consent forms were obtained from all owners. All diagnosed CKD dogs were undergoing blood and urine collections. The clinical data were also retrieved and recorded. The study protocol was conducted in accordance with the standard clinical practice protocols, the animal use guidelines, and the Institutional Animal Care and Use Committee approval (protocol No 2031081).

### Study period and location

The study was conducted from September 2020 to February 2022. The enrolled dogs in this study were client-owned dogs which were treated at the Small Animal Teaching Hospital, Faculty of Veterinary Science, Chulalongkorn University, Thailand.

### Animals and criteria

Experienced veterinarians confirmed the presence of CKD in all 37 client-owned dogs based on medical history, a complete physical examination, radiographic imaging and/or ultrasonography, and laboratory evaluation. The severity of proteinuria in all dogs was categorized into two groups based on the average mean urinary protein–creatinine (UPC) ratio of this study at 4.1 to be UPC ratio <4 and >4 groups, respectively.

### Experimental protocol

All dogs with CKD received a complete physical examination and blood collection on the 1^st^ day of the study. A total of 2.5 mL of blood sample was collected from cephalic or saphenous venipuncture. The 0.5 mL of blood was placed in an ethylenediaminetetraacetic acid tube for complete blood count (CBC) analysis, and another 1 mL of blood was put in a heparinized tube for measurements of blood chemistries (i.e., blood urea nitrogen [BUN]; creatinine [CR]; total protein [TP]; ALB; and inorganic phosphorus [Pi]). Additional 1 mL of blood was separated and placed into plain tubes, allowed to clot, and then centrifuged at 4°C, 1000× *g* for 15 min to separate serum. Approximate 500 μL of serum was kept at –20°C to measure serum concentrations of iron, TF, and UIBC. The urine samples of all dogs were collected by voiding or urinary catheterization and 5 mL of urine was kept at –20°C for measurements of concentrations of protein, iron, TF, and CR.

### Analytical procedure

Hematology was analyzed using an automated machine (BC-5000Vet, MINDRAY, Shenzhen, PR China). The blood biochemistry (BUN, CR, TP, ALB, and Pi) was analyzed using an automated machine (ILAB 650 Chemistry Analyzer, Diamond diagnostics, Holliston, MA, USA). Serum and urine iron concentration and serum UIBC were analyzed by the standard colorimetric ferrozine method (Cobas c501, Roche Diagnostics, Indianapolis, IN, USA). The TIBC was calculated by the sum of plasma iron and UIBC. The TF level in both plasma and urine was analyzed by enzyme-linked immunosorbent assay method (Canine TF ELISA Kit [ab157704], Abcam, Cambridge, UK). The urinary protein was analyzed by multicolor method (Olympus Au 400, Olympus America Inc., Melville, NY, USA). Urinary protein, iron, and TF were divided by urinary CR and expressed as UPC ratio, urinary iron per CR ratio (U-Iron/CR), and urinary TF per CR ratio (U-TF/CR), respectively.

### Statistical analysis

All statistical analyses were performed using SigmaStat Version 12.0 (Systat Software Inc, California, USA). The data are presented as means ± standard error. The differences between proteinuric groups were tested using an unpaired t-test. The correlations among parameters were analyzed by Pearson correlation. A probability value of p < 0.05 was regarded as being statistically significant.

## Results

### Dog characteristics

In this study, 37 dogs with CKD were recruited. No differences in age or weight were found when dogs were divided into two groups based on the severity of proteinuria as UPC ratio <4 group (n = 22) and UPC ratio >4 group (n = 15) (Table-1). Breed, sexual status, and existing comorbidities of dogs in each group varied. Some dogs presented with comorbid diseases as shown in Table-1. In addition, some anemic dogs with CKD received iron, darbepoetin administration, and/or blood transfusion. The average dose of iron was 38.9 mg of element iron/dog/day (range: 6–100 mg of element iron/dog/day), and the duration of administration was 29.6 days (range: 1–60 days). Darbepoetin was injected subcutaneously in some dogs every 1 week at the dose of 1 mg/kg. The duration of darbepoetin treatment varied between 7 and 21 days, and the maximal injection was 3 times. The mean initial HCT in those dogs before darbepoetin was 19.7% ± 1.4%, and it was not different from the day of study (22.9% ± 1.9%). Two dogs received blood transfusion 2 and 3 weeks before the study. The initial HCT before transfusion was 14.8% and 12.8%, whereas HCT after transfusion was 13.0% and 9.7%, respectively.

**Table-1:** Characteristics of dogs categorized by degree of proteinuria.

Parameters	Proteinuria	

	UPC ratio <4 (n = 22)	UPC ratio >4 (n = 15)
Age (years)	11.5 ± 0.9	9.5 ± 1.0
Weight (kg)	11.7 ± 1.6	9.9 ± 2.8
Breed		
Mixed	15	5
Poodle	3	3
Shih tzu	1	1
Pomeranian	1	4
Chihuahua	1	-
Yorkshire terrier	1	-
Beagle	-	1
Bull terrier	-	1
Neutered status		
M/Mc/F/Fs	4/8/1/9	6/1/1/7
Comorbidities		
*E. canis* infectious	5	3
Cardiovascular	2	1
Inflammatory	3	1
Urolith	-	2
Hepatobiliary	1	1
Neurological	3	-
Ophthalmic	-	1
Dermatological	1	-
Treatments		
Iron supplement	7	9
Darbepoetin injection	6	6
Blood transfusion	1	1

Data are presented as mean ± standard error. UPC=Urinary protein–creatinine, M=Male, Mc=Castrate male, F=Female, Fs=Spayed female, *E. canis=Ehrlichia canis*

### Blood profiles in dogs as categorized by severity of CKD

The number of dogs in Stages I, II, III, and IV CKD as categorized by International Renal Interest Society (IRIS) group were 5, 11, 13, and 8, respectively. The CBC and some biochemical profiles are shown in Table-2. The HCT was reduced along with the severity of CKD. Stage IV CKD had significantly lower HCT compared with Stage I (p < 0.05). The BUN and Pi were significantly higher when renal impairment was advanced. Lower TP was found in Stage III compared with Stage I (p < 0.05). The ALB tended to be reduced without significance along with the severity of CKD. The white blood cell, alanine transferase, and UPC ratio were not different among stages.

**Table-2:** The complete blood count and blood chemistries in all dogs as categorized by stage of CKD.

Parameters	n	Stage of CKD			

		I	II	III	IV
HCT (%)	5/11/13/8	37.4 ± 2.8^a^	29.0 ± 3.3^ab^	26.7 ± 5.1^ab^	18.5 ± 2.3^b^
WBC (×10^3^ cells/mL)	5/11/12/8	7.23 ± 1.86	14.69 ± 3.59	9.92 ± 2.52	13.27 ± 4.02
BUN (mg/dL)	5/11/13/8	47.3 ± 10.9^bc^	48.2 ± 6.8^b^	92.6 ± 23.0^ac^	158.2 ± 28.0^a^
CR (mg/dL)	5/11/13/8	0.96 ± 0.17^b^	2.14 ± 0.10^b^	3.99 ± 0.42^ac^	8.49 ± 0.71^a^
ALT (U/L)	4/10/11/8	129.5 ± 51.3	108.9 ± 18.4	54.1 ± 32.5	117.4 ± 32.0
TP (g/dL)	5/11/13/8	7.82 ± 0.53^a^	6.68 ± 0.35^ab^	6.20 ± 0.62^b^	6.86 ± 0.27^ab^
ALB (g/dL)	5/11/13/8	2.50 ± 0.27	2.34 ± 0.11	2.14 ± 0.18	2.05 ± 0.16
Pi (mg/dL)	5/10/13/7	3.7 ± 0.3^b^	5.2 ± 0.6^b^	9.6 ± 1.2^ac^	12.4 ± 1.8^a^
UPC ratio	5/11/13/8	3.32 ± 1.37	2.82 ± 0.79	5.94 ± 2.58	3.28 ± 0.72

Data are presented as mean ± standard error. Different superscripts indicate a significant difference (p < 0.05). CKD=Chronic kidney disease, HCT=Hematocrit, WBC=White blood cell, BUN=Blood urea nitrogen, CR=Plasma creatinine, ALT=Alanine transferase, TP=Total protein, ALB=Albumin, Pi=Inorganic phosphorus, UPC=Urinary protein–creatinine

### Blood profiles and iron parameters in dogs as categorized by degree of proteinuria

The blood parameters did not differ between groups when dogs were categorized based on proteinuria (Table-3). However, TIBC was significantly lower (p < 0.001) while U-Iron/CR and U-TF/CR were significantly higher in UPC ratio >4 group than those in UPC ratio <4 group.

**Table-3:** The blood and urinary parameters in dogs with different degrees of proteinuria.

Parameters	n	Degree of proteinuria	

		UPC ratio <4	UPC ratio >4
HCT (%)	22/15	28.8 ± 2.4	24.6 ± 2.1
WBC (×10^3^ cells/mL)	21/15	11.67 ± 1.69	11.86 ± 2.78
BUN (mg/dL)	22/15	91.7 ± 15.5	81.3 ± 10.2
CR (mg/dL)	22/15	4.1 ± 0.7	3.8 ± 0.4
ALT (U/L)	20/13	112.5 ± 17.5	68.5 ± 16.1
TP (g/dL)	22/15	6.9 ± 0.2	6.4 ± 0.3
ALB (g/dL)	22/15	2.27 ± 0.10	2.17 ± 0.07
Pi (mg/dL)	20/15	7.3 ± 1.1	9.1 ± 0.7
Iron (mg/dL)	22/15	114.5 ± 9.4	101.1 ± 12.9
TIBC (mg/dL)	22/15	339.8 ± 19.9	231.3 ± 19.9***
TF (mg/dL)	14/14	448.7 ± 55.2	366.8 ± 36.6
U-Iron/CR (mg/mg CR)	22/15	246.9 ± 37.0	457.1 ± 84.6**
U-TF/CR (mg/mg CR)	14/14	25.8 ± 6.0	73.7 ± 9.5***
UPC ratio	22/15	2.0 ± 0.1	7.2 ± 0.9***

Data are presented as mean ± standard error.

** =p < 0.01,

*** =p < 0.001 compared to UPC ratio <4 group. n=Number of dogs

(UPC ratio <4/UPC ratio >4), HCT=Hematocrit, WBC=White blood cell, BUN=Blood urea nitrogen, CR=Plasma creatinine, ALT=Alanine transferase, TP=Total protein, ALB=Albumin, Pi=Inorganic phosphorus, TIBC=Total iron-binding capacity, TF=Plasma transferrin, U-Iron/CR=Urinary iron per creatinine ratio, U-TF/CR=Urinary transferrin per creatinine ratio, UPC=Urinary protein–creatinine

### Relationship among parameters

**Figure-1:**
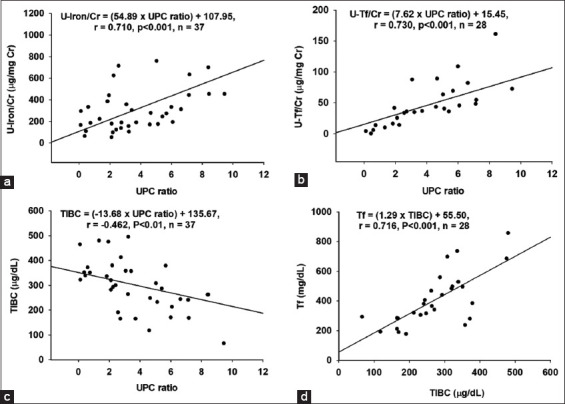
Relationships between (a) Urinary protein–creatinine ratio and urinary iron per creatinine ratio, (b) Urinary transferrin (TF) per creatinine ratio, (c), Total iron-binding capacity (TIBC), and (d) Relationship between plasma TF and TIBC.

HCT was correlated negatively with BUN (p < 0.01), CR (p < 0.001), and Pi (p < 0.01) but positively with ALB (p < 0.05) (Table-4). Strong positive relationships were found between UPC ratio and U-Iron/CR (p < 0.001) (Figure-1a) and U-TF/CR (p < 0.001) (Figure-1b) but negative with TIBC (p < 0.01) (Figure-1c). The TIBC was correlated positively with both ALB (p < 0.05) and plasma TF concentration (p < 0.001) (Figure-1d). Urinary iron per CR ratio was correlated positively with U-TF/CR (p < 0.01).

**Table-4:** Correlation coefficients among blood chemistries, iron parameters, and UPC ratio.

Parameters	ALB	HCT	BUN	CR	Pi	Iron	TIBC	TF	U-Iron/CR	U-TF/CR
UPC ratio	−0.117	−0.229	0.045	0.008	0.265	−0.124	−0.462**	−0.276	0.710***	0.730***
ALB		0.415*	−0.202	−0.282	−0.126	−0.028	0.408*	0.299	−0.305	−0.142
HCT			−0.482**	−0.550***	−0.482**	0.025	0.166	−0.132	−0.294	−0.012
BUN				0.793***	0.795***	0.199	0.019	0.034	0.122	−0.144
CR					0.786***	0.077	0.015	0.141	0.065	−0.137
Iron							0.150	−0.020	0.099	−0.143
TIBC								0.716***	−0.203	−0.293
TF									−0.005	−0.291
U-Iron/CR										0.486**

* p < 0.05;

** p < 0.01;

*** p < 0.001. UPC ratio=Urinary protein–creatinine ratio, ALB=Albumin, HCT=Hematocrit, BUN=Blood urea nitrogen, CR=Creatinine, PI=Inorganic phosphorus, TIBC=Total iron-binding capacity, TF=Transferrin, U-Iron/CR=Urinary iron per creatinine ratio, U-TF/CR=Urinary transferrin per creatinine ratio

## Discussion

In this study, the dogs developed CKD with proteinuria. Both groups with low and high UPC ratios included a variety of breeds with different sexual status and were old. The prevalence of CKD was found in old age, which was due to renal impairment over time. Rather than old age, risk factors for CKD development were inflammatory/infectious diseases, history of anesthetic-surgical procedures, heart disease, neoplasm, endocrinopathies, and exposure to nephrotoxic drugs [2]. Canine vector-borne disease, particularly *Ehrlichia* spp., was associated with proteinuria [19]. Dogs with *E. canis* infection had membranoproliferative glomerulopathy and interstitial nephritis along with hypoalbuminemia [20]. In case of heart disease, three dogs had degenerative mitral valve disease, American College of Veterinary Internal Medicine stage B1 and not receiving any diuretic drugs. All dogs are in stable hydration status. Two dogs had urinary calculi. One dog had nephrolithiasis, and the other had cystic calculi. The calculi were not removed, but no urinary tract obstruction and infection were found. Many dogs received drugs to treat their concurrent diseases. However, the dose and duration of treatment were in a standard recommendation that should not affect kidney function. Some dogs had anemia and required iron supplementation, darbepoetin administration, or blood transfusion.

Both degrees of renal impairment and proteinuria are important factors determining survival in patients with CKD. In dogs, UPC ratio >0.5 was significantly associated with reduced survival and proteinuria, which could increase the risk of death at any time point [1]. The previous study in cats with CKD showed that both plasma CR and UPC ratio were associated with shorter renal survival time [3, 4]. In dogs, slow disease progression and higher survival rates were related to persistent monitoring of SDMA, renal proteinuria, and timely therapeutic management [2]. The IRIS group categorized the severity of kidney disease and proteinuria in which the upper value of plasma CR in Stage III was 5.0 mg/dL, whereas proteinuria in dogs is considered when the UPC ratio is higher than 0.5 [21]. Most of the dogs in this study (33/37) had UPC ratio higher than 0.5.

Besides proteinuria, another risk factor related to decreased survival rate is anemia [2]. In patients with diabetic nephropathy, after clinical and renal pathologic covariate adjustment, anemia was associated with adverse renal outcomes [22]. Increased urea, phosphate, and decreased HCT were dependent risk factors that were associated with shorter renal survival time in cats with CKD [4]. The present study showed that the HCT of dogs was related to the severity of renal impairment as categorized by the stage of CKD in which higher CKD stage had more anemia. Hematocrit and renal function parameters showed a negative correlation (BUN; p < 0.01, CR; p < 0.001 and Pi; p < 0.01). The results were similar to the previous study in dogs with CKD [12]. The reduction in HCT, along with severity of CKD was mainly due to the failure to produce EPO relative to the severity of anemia [5]. In addition, desensitization of the oxygen-sensing mechanism in EPO-producing cells by uremic toxin indoxyl sulfate was demonstrated in human hepatoma cell line HepG2 [6].

Other than a lack of EPO in CKD, other factors may contribute to anemia in patients with CKD. Plasma ALB concentration was negatively correlated with HCT. The results were similar to the previous study in CKD and control healthy dogs [14]. This association was also found in patients with diabetic nephrosclerosis [23]. Decreased ALB in CKD may be associated with reduced production from anorexia or increased urinary loss in protein losing nephropathy. Therefore, proteinuria may aggravate the progression of CKD by reduced ALB and other factors contributing to erythropoiesis, including iron, TF, EPO, transcobalamin, and/or copper [17]. Thus, heavy proteinuria could alter erythrocyte metabolism resulting in enhanced erythrocyte death and anemia [24]. Moreover, many anemic dogs with CKD failed to respond to the EPO treatment and iron supplementation [13, 14]. It is likely that iron metabolism is one of important factors affecting anemia in patients with CKD. The iron balance is regulated by dietary iron absorption and iron sequestration from storage sites, such as liver and reticuloendothelial macrophages. Among iron parameters, TIBC is a reliable indicator of iron metabolism. The iron content when TF is saturated with iron is TIBC. Total iron-binding capacity could indirectly indicate plasma TF level, which was confirmed by a strong positive relationship between TF and TIBC in the present study. Some studies recommended TF determination using immunologic-based techniques rather than TIBC, although genetic variation in TF was found in some populations [25]. The level of iron in dogs of all groups was lower than those measured from control healthy dogs [14]. The TIBC was lower in UPC ratio >4 group than UPC ratio <4 groups, and TIBC was negatively correlated with UPC ratio. Thus, TIBC was affected by the degree of proteinuria. Lower TIBC and HCT were found in both dogs and cats with CKD than in healthy control [14, 26]. Unfortunately, proteinuria was not evaluated in both studies.

Iron deficiency can be characterized as a true iron deficiency when serum iron and ferritin concentrations are low and TIBC is high. In contrast, in cases of functional iron deficiency, ferritin concentrations are generally normal or above the reference interval, and serum iron concentrations and TIBC are generally decreased [11]. Functional iron deficiency was suggested in CKD cats, in which mean total iron and TIBC were lower than control healthy cats with no alteration of ferritin level [26]. The same may be applied to dogs with CKD, especially those with proteinuria. Unfortunately, plasma ferritin was not measured in this study.

The present study showed that dogs with CKD with proteinuria had a urinary loss of iron and TF, resulting in a lower TIBC. The UPC ratio was correlated positively with both U-Iron/CR and U-TF/CR and negatively with TIBC. Excessive urinary losses of iron, TF, EPO, transcobalamin, and some metals were found in patients with nephrotic syndrome [17]. In humans with diabetic nephropathy, the urinary ALB was related to urinary TF, and urinary TF significantly increased with respect to the progression of glomerular diffuse lesions [27]. Higher urinary loss of TF than ALB was found due to higher isoelectric point of TF in which less polyanion may favor the higher excretion, although TF has slightly higher molecular weight than ALB (77 kD vs. 66 kD) [28]. The urinary TF was suggested to be used as a diagnostic marker of early diagnosis of renal disease in Stage I of CKD cat since leakage of urinary TF in urine precedes leakage of urinary ALB, and their sensitivity and specificity were higher than those of plasma CR concentration [18].

The U-Iron/CR and U-TF/CR were also correlated (r = 0.486, p < 0.01, n = 28). Urinary iron excretion was found to be associated with urinary TF and urinary protein in CKD human patients [29]. In a patient with overt proteinuria, iron/TF ratio in urine was greater than the ratio in plasma, suggesting that renal handling of iron may be dissociated from TF [30]. The inconsistent rate of excretion may be due to different rates of filtration, tubular reabsorption, and the existence of dissociated species of iron and TF that could be filtered.

This study cannot rule out the factors affecting some parameters, such as inflammation or nutritional status. Inflammatory cytokines were associated with iron metabolism and red blood cell profiles in patients with CKD [31]. Inflammatory cytokines can result in anemia by decreased production of endogenous EPO, delayed response of erythroid progenitor to EPO, and increased production of hepcidin which was extensively reviewed [8, 32, 33]. During the acute phase response of non-specific inflammatory reaction of the host, some negative acute phase proteins (APPs), such as ALB and TF, were reduced, while some positive APPs, such as serum amyloid A, were increased [34]. Furthermore, hepcidin, the iron regulatory hormone, which can be induced by inflammation and contributes to anemia was documented [7, 11, 35–38]. In cats with CKD, the mean serum iron concentration, TIBC, and HCT were lower, whereas serum amyloid A and hepcidin levels were higher than in healthy control cats [26]. However, no change in hepcidin was found in dogs with CKD [14]. Rather than inflammation, nutritional status can affect plasma TF concentration. A report on using TF as a nutritional marker for malnutrition in dogs receiving nutritional treatment was documented [39]. It is possible that the lower TF in this study may be in part due to the malnourishment of dogs with CKD.

In this study, anemia may be associated with external factors other than the degree of CKD and proteinuria. Anemia during the acute phase of naturally infected *E. canis* was reported to be associated with altered oxidative status, low iron levels, and high levels of both ferritin and TF [40]. Supplementation of iron had no effect on HCT. Similar results were found in dogs with CKD in which iron supplementation did not affect HCT, plasma iron levels, and TIBC [14]. In addition, darbepoetin administration did not relate to HCT level. The maximum number of darbepoetin administration was three injections in five dogs. The HCT was unaltered after injection, which may be due to the duration after injection until blood collection was too short, or dogs did not respond to darbepoetin. It was reported that median time to achieve an HCT ≥30% was 29 days after administration with a dose of 0.4–2.1 μg/kg given once a week [13]. The low response to darbepoetin treatment and iron supplements may also be a result of erythroid hypoplasia, which was commonly reported in dogs with CKD in varying stages [12]. This bone marrow feature could also affect erythropoiesis and HCT. However, bone marrow aspiration was not performed in this study. Finally, blood transfusion was not related to HCT in the present study. The HCT was declined quickly within a few weeks after transfusion.

This study has some limitations. The number of dogs with CKD in the present study was small. Moreover, the inflammatory cytokines, ferritin, EPO levels, and bone marrow status were not measured.

## Conclusion

High urinary loss of iron and TF along with low TIBC was associated with a degree of proteinuria and may be a contributing factor of anemia in dogs with CKD. Reduced urinary loss and/or supplementation of iron may be helpful in this situation.

## Authors’ Contributions

CB, NS, and SS: Conception and design of the study, statistical analysis, and drafted the manuscript. NS, MT, and TD: Data collection and laboratory analysis. CB, NS, and SS: Revised the manuscript. All authors have read and approved the final manuscript.
